# Non-autocrine, constitutive activation of Met in human anaplastic thyroid carcinoma cells in culture

**DOI:** 10.1038/sj.bjc.6690406

**Published:** 1999-05

**Authors:** J D Bergström, A Hermansson, T Diaz de Ståhl, N-E Heldin

**Affiliations:** Department of Genetics and Pathology, Unit of Pathology, University Hospital, Uppsala, S-751 85, Sweden

**Keywords:** Met, thyroid, anaplastic carcinoma, receptor activation

## Abstract

Activation of Met by its ligand HGF has been shown to elicit both mitogenic and motogenic responses in thyrocytes in vitro. In the present study we have investigated the expression of Met in human anaplastic thyroid carcinoma cells in culture. There was a variation in expression level and size of Met in the different cell lines; high Met expression was found in four cell lines, compared to non-neoplastic human thyrocytes. Treatment with glucoproteinase F showed that the size differences observed were due to variances in the degree of glycosylation. Interestingly, in cell lines with high expression of Met, the receptor proteins were found to be constitutively tyrosine phosphorylated. None of these cell lines expressed HGF mRNA, and addition of suramin did not affect the level of tyrosine phosphorylation of Met in unstimulated cells, suggesting the absence of autocrine stimulatory pathways. Furthermore, we did not observe *MET* gene amplification, activating mutations or phosphatase defects. The tyrosine phosphorylated receptors appeared functionally active since the receptors associated with the adaptor molecule Shc. In summary, we have found ligand-independent constitutively activated Met in four out of six anaplastic thyroid carcinoma cell lines. © 1999 Cancer Research Campaign


*British Journal of Cancer* (1999) **80**(5/6), 650–656
© 1999 Cancer Research Campaign
Article no. bjoc.1998.0406

### Non-autocrine, constitutive activation of Met in human anaplastic thyroid carcinoma cells in culture

**JD Bergström, A Hermansson, T Diaz de Ståhl and N-E Heldin**

Department of Genetics and Pathology, Unit of Pathology, University Hospital, S-751 85 Uppsala, Sweden

**Summary** Activation of Met by its ligand HGF has been shown to elicit both mitogenic and motogenic responses in thyrocytes in vitro. In the present study we have investigated the expression of Met in human anaplastic thyroid carcinoma cells in culture. There was a variation in expression level and size of Met in the different cell lines; high Met expression was found in four cell lines, compared to non-neoplastic human thyrocytes. Treatment with glucoproteinase F showed that the size differences observed were due to variances in the degree of glycosylation. Interestingly, in cell lines with high expression of Met, the receptor proteins were found to be constitutively tyrosine phosphorylated. None of these cell lines expressed HGF mRNA, and addition of suramin did not affect the level of tyrosine phosphorylation of Met in unstimulated cells, suggesting the absence of autocrine stimulatory pathways. Furthermore, we did not observe *MET* gene amplification, activating mutations or phosphatase defects. The tyrosine phosphorylated receptors appeared functionally active since the receptors associated with the adaptor molecule Shc. In summary, we have found ligand-independent constitutively activated Met in four out of six anaplastic thyroid carcinoma cell lines.

**Keywords:** Met; thyroid; anaplastic carcinoma; receptor activation

The receptor tyrosine kinase Met consists of two disulphide-linked polypeptide chains, α and β, with molecular masses of 50 and 145 kDa respectively (Giordano et al, 1989). Hepatocyte growth factor (HGF), also known as scatter factor (SF), has been demonstrated to be the ligand for Met (Bottaro et al, 1991; Naldini et al, 1991). Met is expressed in a variety of cell types, mainly of epithelial origin (Zarnegar and Michalopoulos, 1995). Activation results in a number of cellular responses, such as growth, motility and morphogenesis, depending on cell type (Stoker et al, 1987; Higashio et al, 1990; Rubin et al, 1991; Weidner et al, 1991). In the cytoplasmic domain of the receptor protein there are two tyrosine phosphorylation sites (Y1234 and Y1235) needed for activation of the kinase domain (Longati et al, 1994; Zhen et al, 1994), and two others for association with different SH2-domain containing signalling proteins (Y1349 and Y1356) (Ponzetto et al, 1994). Met has been shown to interact with Grb2, phospholipase C-γ (PLC-γ), phosphoinositol (PI) 3-kinase, Shc and Src (Bardelli et al, 1992, 1994), as well as Gab 1 (Weidner et al, 1996) and a tyrosine phosphatase (SHP2/Syp 2) (Villa-Moruzzi et al, 1993; Fixman et al, 1996). Recently, it was demonstrated that a member of the ets family of transcription factors, ets 1, positively regulates the expression of Met (Gambarotta et al, 1996).

Overexpression of Met has been reported in several different types of carcinomas, including ovarian (Di Renzo et al, 1994), pancreatic (Di Renzo et al, 1995*a*), colorectal (Di Renzo et al, 1991; Prat et al, 1991) and thyroid carcinomas (Di Renzo et al, 1991, 1992, 1995*b*). High expression of Met protein was found in approximately 70% of papillary thyroid carcinomas. The overexpression was not related to gene amplification, but the histotype of the tumours correlated to bad prognosis for the patients (Di Renzo et al, 1992). In normal thyroid epithelium in vivo Met is only expressed at a very low level (Di Renzo et al, 1991, 1992). However, HGF has been shown to be a very potent mitogen for both human and dog thyroid follicle cells (Dremier et al, 1994; Eccles et al, 1996). Besides the growth stimulatory effect, HGF also induced de-differentiation of the thyrocytes, as have been observed after stimulation with other thyroid mitogens (Westermark et al, 1983). Furthermore, expression of HGF has also been found within the human thyroid (Zarnegar et al, 1990), making a paracrine stimulation possible in the regulation of thyroid follicle cell growth and function.

In the present study we have investigated the expression and activity of Met in human anaplastic thyroid carcinoma cell lines, as well as in non-neoplastic human thyrocytes in primary culture. We found an overexpression of Met in a majority of the carcinoma cell lines, viz. in HTh 83, C 643, SW 1736 and KAT-4, compared to the level found in normal thyrocytes. In these thyroid carcinomas, Met appeared to be constitutively activated in a ligand-independent fashion.

### MATERIALS AND METHODS

### Cell lines

The human anaplastic thyroid carcinoma cell lines HTh 7 (Carlsson et al, 1983), HTh 74 (Heldin et al, 1991), HTh 83 (Dahlman et al, unpublished results), C 643 (Heldin et al, 1988), SW 1736 and KAT-4 (Ain and Taylor, 1994) were routinely grown in Eagle's minimal essential medium (HTh 7, HTh 74, C 643 and SW 1736) or in RPMI 1640 medium (HTh 83 and KAT-4). As a control for Met expression we used a hepatic carcinoma cell line, Hep G2, cultured in RPMI 1640 medium. The B-cell line U2889, used as a control in the Southern blot analysis, was cultured in RPMI 1640 medium. JJN 3 (Börset et al, 1996), a myeloma cell

*Received 17 April 1998*
*Revised 20 October 1998*
*Accepted 23 December 1998*

*Correspondence to:* JD Bergström line used as positive control in the HGF Northern blot analysis, was also cultured in RPMI 1640 medium. All media were supplemented with 10% calf serum and antibiotics (100 U of penicillin and 50 µg of streptomycin per ml). The primary cultures of human thyroid follicle cells were established by collagenase treatment of non-neoplastic thyroid tissue (Heldin and Westermark, 1988), and cultured in F12 medium containing 1% calf serum and antibiotics.

### Northern blot analysis

Total RNA was extracted from cells using a LiCl/urea method (Auffray and Rougeon, 1980), and mRNA was enriched by poly-$A^+$ selection. Total or poly-$A^+$ RNA (15 or 10 µg per lane respectively) was size-fractionated in a 0.8% agarose gel under denaturating conditions and transferred to a nitrocellulose filter. The filters were hybridized as described before (Heldin and Westermark, 1988), using a $^{32}P$-labelled (Megaprime kit; Amersham Pharmacia Biotech, Uppsala, Sweden) human Met (Rong et al, 1992) or a 0.7 kb human HGF-β subunit cDNA fragment (pKK233-DEβ5; Nakamura et al, 1989). The hybridization with the HGF probe was performed using QuikHyb solution (Stratagene, La Jolla, CA, USA). After a wash in 2 × saline sodium citrate (SSC) (1 × SSC consists of 0.15 M sodium chloride and 0.125 M sodium citrate, pH 7.0) and 0.5% sodium dodecyl sulphate (SDS) for 30 min at 37°C, and a subsequent wash in 0.1 × SSC and 0.1% SDS at 60°C for 25 min. Filters were autoradiographed at – 70°C using Kodak XAR-5 films and intensifying screens. In order to check for loading differences the filters were hybridized with a glyceraldehyde 3-phosphate dehydrogenase (GAPDH; pGAP) probe (Tso et al, 1985).

### Southern blot analysis

Chromosomal DNA was extracted from the cell lines was incubated with proteinase K (100 µg $ml^{-1}$) in 10 mM Tris–HCl, 1 mM EDTA, 1% SDS and 0.1 M sodium chloride (NaCl) for 20 h at 37°C, followed by a phenol extraction. Chromosomal DNA was precipitated in ethanol after extraction twice with isobutanol/isopropanol (7:3). Chromosomal DNA was digested for 16 h at 37°C with *Eco*RI and samples were electrophoresed and transferred to a nitrocellulose filter using standard methods. The filter was hybridized and autoradiographed as described above (Heldin and Westermark, 1988). As a control for equal loading, the filter was stripped and rehybridized with a 3 kb human PAI-1 c DNA fragment (pPAI3; obtained from Dr Sawdey, Scripps Research Institute, CA, USA).

### Western blot analysis

Cells were incubated with or without HGF (rhHGF; R&D Systems, Oxford, UK), 10 ng $ml^{-1}$, for 6 min at 37°C, washed with phosphate-buffered saline (PBS) containing 1 mM $Na_3VO_4$, and lysed in a buffer consisting of 1% Triton X-100, 150 mM NaCl, 10 mM Tris–HCl pH 7.4, 1 mM EGTA, 1 mM EDTA and 0.5% NP-40, with protease and phosphatase inhibitors (35 ng $ml^{-1}$ phenylmethylsulphonyl fluoride (PMSF), 1.4 µg $ml^{-1}$ aprotinin, 1 mM $Na_3VO_4$, 10 mM NaF, 1 mM $ZnCl_2$ and 50 µM $Na_2MoO_4$). After a 30 min incubation on ice the lysates were centrifuged for 15 min at 20 000 ***g*** at 4°C. The clear supernatants were collected and used in the experiments. The protein concentrations were determined using a commercial kit (Pierce Chemical Co., Rockford, IL, USA). Following SDS-polyacrylamide gel electrophoresis (SDS-PAGE) under reducing or non-reducing conditions, an immunoblot analysis was performed using anti-Met (C-28) or anti-Syp 2 antibodies (C-18, Santa Cruz Biotechnologies, Santa Cruz, CA, USA), with a secondary horse-radish peroxidase conjugated anti-rabbit-IgG (Amersham Pharmacia Biotech, Uppsala, Sweden). Detection of signals was done with a commercial kit for chemoluminescense (Pierce Chemical Co., Rockford, IL, USA).

### Glycoproteinase F digestion

Total cell lysate (20 µg per sample) from each cell line was diluted to 100 µl with a buffer consisting of 0.25 M $Na_2HPO_4$, pH 7.5, 10 mM EDTA, 10 mM β-mercaptoethanol (β-ME) and 0.5% (w/v) n-octylglucoside, boiled 3 min and digested with 2 U glycoproteinase F (USB, Cleveland, OH, USA) for 24 h at 37°C. Samples were analysed in a Western blot using the anti-Met antibody.

### Immunoprecipitation of tyrosine phosphorylated proteins

Cell lysates were prepared as described above. Tyrosine phosphorylated proteins were immunoprecipitated from 0.5 to 1 mg of total lysate (0.5 mg for the experiment in Figure 4 and 1.0 mg for experiment in Figure 5) using agarose-conjugated anti-phosphotyrosine (PY) antibodies (PY-20-agarose; Transduction Laboratories, Lexington, KY, USA). Lysates were incubated for 1.5 h at 4°C, and subsequently washed four times in lysis buffer. The samples were boiled in sample buffer containing β-ME, and separated on a 7.5% polyacrylamide gel. Immunoblots with the anti-Met antibody were done as previously described.

### Co-immunoprecipitation of Met and Shc proteins

Total cell lysates (1.2 mg per sample) were incubated with anti-Shc antibodies (1 µg $ml^{-1}$; PG-797 from Santa Cruz Biotechnologies, Santa Cruz, CA, USA) for 1 h at 4°C. After another 45 min incubation with GammaBind Plus Sepharose (Amersham Pharmacia

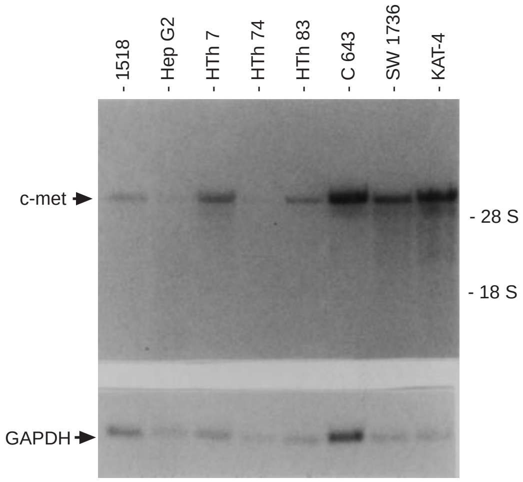

**Figure 1** Expression of c-*met* mRNA in human anaplastic thyroid carcinoma cell lines. Ten micrograms of poly $A^+$ -RNA were separated in a 0.8% agarose gel, transferred to a nitrocellulose filter and hybridized with a human Met cDNA probe as described in Materials and Methods

*© Cancer Research Campaign 1999*

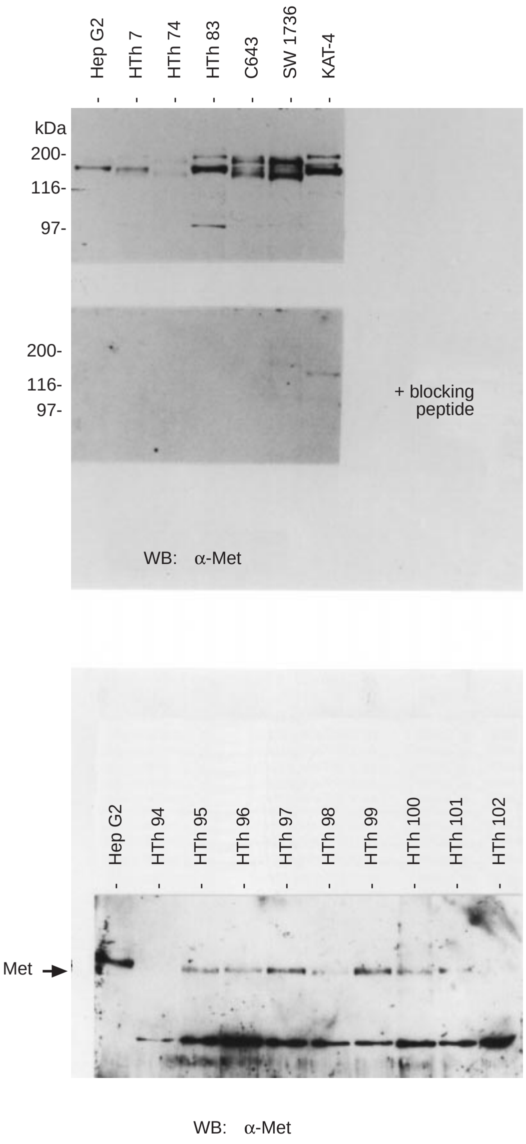

**Figure 2** Met protein level in anaplastic thyroid carcinoma cell lines and primary cultures of non-neoplastic human thyroid follicle cells. (**A**) Total cell lysates (10 μg per lane) from the thyroid carcinoma cell lines were separated by SDS-PAGE and transferred to a nitrocellulose filter and immunoblotted as described in Materials and Methods using an anti-Met antibody. To detect the specificity of the bands observed, the antibody was blocked with the immunizing peptide prior to the immunoblotting (the lower part of panel **A**). (**B**) Total lysates from primary cultures of non-neoplastic human thyrocytes were immunoblotted as described above with the anti-Met antibody. In order to detect Met in total lysates from the primary cultures a prolonged exposure had to be performed. The amount loaded of Hep G2 lysate was the same in panels **A** and **B**

Biotech, Uppsala, Sweden) the precipitates were washed three times in lysis buffer, boiled in SDS-PAGE sample buffer with β-ME, and Western blots were performed as described above using the anti-Met antibody.

### RT-PCR and sequencing

Total RNA was extracted from the HTh 83 and KAT-4 cells as described above. The RNA was converted into cDNA using the forward polymerase chain reaction (PCR) primer as template, utilizing a commercial kit (GeneAmp, Perkin-Elmer, NJ, USA). The cDNA was amplified by PCR, 30 cycles, using two primers spanning exon 18 and 19 of the *MET* gene: GGCAA-GAAAATTCACTGTGC (forward) and TCCCAGAGGAC-GACGCCAAA (reverse). Following PCR, the reaction products were purified using a commercial kit (Qiaquick PCR-purification kit, Qiagen, Hilden, Germany) and ligated into the pGEM-T cloning vector (Promega, Madison, WI, USA). The Met fragments were sequenced on an ABI 377 automated sequencer, using the forward and reverse primers described above as sequencing primers.

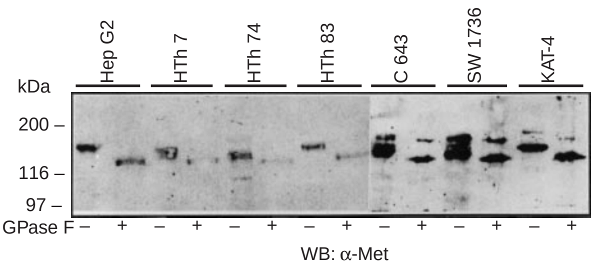

**Figure 3** Glycoproteinase F digestion of Met in the thyroid carcinoma cell lines. To study the size differences of Met, 20 μg of total lysate from each cell line was treated with glycoproteinase F for 24 h to trim off sugar residues. Untreated and glycoproteinase F treated lysates (3 μg per sample) were separated by SDS-PAGE and the filter was immunoblotted with the anti-Met antibody

### RESULTS

### Expression of Met in human anaplastic thyroid carcinoma cell lines

Northern blot analysis of poly-$A^+$-RNA extracted from the anaplastic thyroid carcinoma cells revealed *c-met* transcripts in all six cell lines (Figure 1). The size of the mRNA found was approximately 7 kb, i.e. of the same size as in AG1518 human foreskin fibroblasts and in the hepatic carcinoma cell line Hep G2. The amount of Met mRNA observed differed between cell lines; however, there was a fairly good correlation between the mRNA level and the amount of Met protein detected in immunoblotting experiments (Figure 2A); showing a high or very high expression of Met in HTh 83, C 643, SW 1736 and KAT-4 cell lines, compared to the Met protein level in primary cultures of non-neoplastic human thyroid follicle cells (Figure 2B).

The size of the Met protein varied in the different cell lines. This appeared to be due to differences in glycosylation since treatment with glycoproteinase F resulted in deglycosylated Met proteins of similar size (Figure 3).

The high expression of Met observed was not a result of gene amplification since Southern blot analysis of DNA extracted from the cell lines did not show any signs of increased *MET* gene copy number (Figure 4). As a DNA loading control the filter was hybridized with a plasminogen activator inhibitor-1 (PAI-1) cDNA probe with similar results (data not shown).

### Constitutive tyrosine phosphorylation of Met

The expressed Met proteins seemed functionally active since stimulation with HGF induced tyrosine phosphorylation of the receptors, determined by immunoprecipitation with anti-PY antibodies

© Cancer Research Campaign 1999

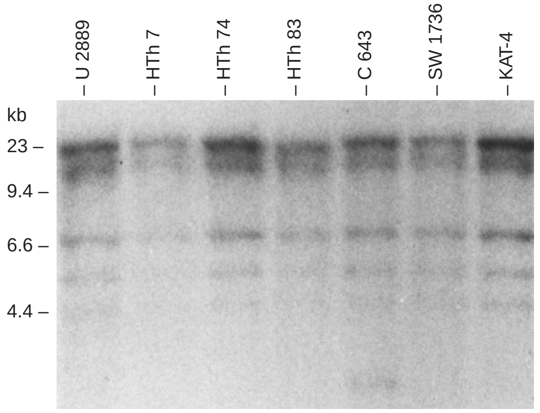

**Figure 4** Southern blot analysis of DNA from the anaplastic thyroid carcinoma cell lines. Chromosomal DNA extracted from the cell lines were subjected to a *Eco* R1 digestion for 16 h at 37°C (10 µg per sample). Southern blot analysis was performed as described in Materials and Methods, using a $^{32}$P-radiolabelled human *MET* cDNA fragment as probe. As normal control we used a human EBV-immortalized B-cell line (U2889)

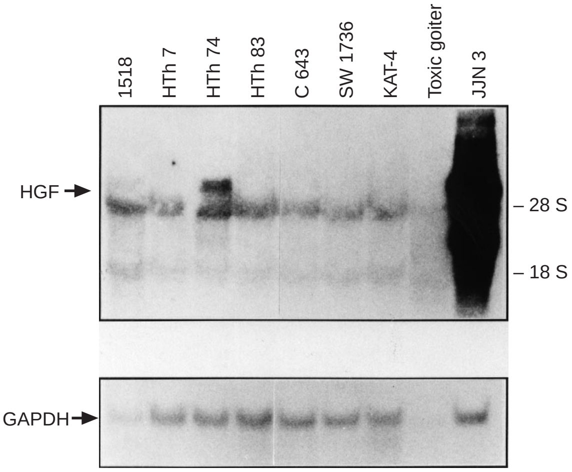

**Figure 6** Expression of HGF mRNA in human anaplastic thyroid carcinoma cell lines. Total RNA (15 µg per lane) was size-fractionated under denaturing conditions by electrophoresis. Following transfer, the filter was hybridized with a human HGF cDNA probe as described in Materials and Methods. The human myeloma cell line JJN 3, known to produce HGF (Börset et al, 1996), was used as a positive control

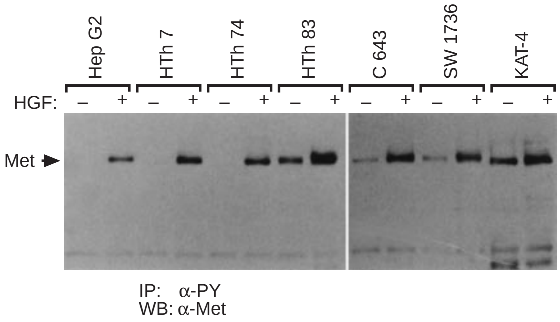

**Figure 5** Immunoprecipitation of Met with a phosphotyrosine antibody (PY-20). Total cell lysates of unstimulated and HGF-stimulated (10 ng ml$^{-1}$, 6 min at 37°C) cells were immunoprecipitated with an anti-PY mAb (PY-20) as described in Materials and Methods. The precipitates were separated on an SDS-PAGE gel and immunoblotted with the anti-Met antibody

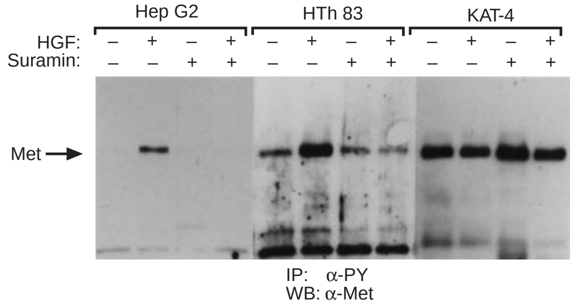

**Figure 7** Effect of suramin on the constitutive activation of Met in thyroid carcinoma cells. Hep G2, HTh 83 and KAT-4 cells were incubated 4 h with suramin (200 µg ml$^{-1}$), and finally incubated with or without HGF (10 ng ml$^{-1}$) for another 6 min. The cells were lysed and total lysates were immunoprecipitated with anti-PY mAb (PY-20), separated on SDS-PAGE and immunoblotted using the anti-Met antibody

(Figure 5). However, in four of the cell lines, HTh 83, C 643, SW 1736 and KAT-4 cells, we found phosphorylated Met also in unstimulated cells. This finding suggests a constitutive phosphorylation and maybe also activation of Met. As seen in Figure 5, in KAT-4 cells there was virtually no difference between the amounts of phosphorylated Met precipitated in unstimulated compared to the HGF-stimulated cells.

To study if the observed tyrosine phosphorylation of Met was due to endogenous production and autocrine stimulation by HGF, the HGF expression in the cell lines was investigated. Northern blot analysis of total RNA extracted from the cell lines showed expression of HGF mRNA only in the HTh 74 cells, and not in the cell lines with constitutively phosphorylated Met proteins (Figure 6). Furthermore, incubation of cells with suramin (200 µg ml$^{-1}$) for 4 h prior to lysis showed the receptors to be tyrosine phosphorylated even in the presence of suramin (Figure 7; only results from HTh 83 and KAT-4 are shown, however, similar results were obtained with the C 643 and SW 1736 cells). Since suramin blocked Met activation by 10 ng ml$^{-1}$ of HGF added to the Hep G2 cells (Figure 7), the current findings suggest a ligand-independent phosphorylation of the receptor in the carcinomas.

Changes or differences in the phosphatase activity might contribute to the constitutive activation of Met found. Therefore, we performed Western blot analysis of the phosphatase functionally coupled to Met, Syp 2, and also compared the phosphatase activity in the different cell lines. Syp 2 protein was found in equal amounts in all cell lines, and we could not detect any differences in the phosphatase activity (data not shown). Moreover, PCR-amplification and nucleotide sequencing of exon 17–19 of the kinase domain of the *MET* gene, known to contain mutations in renal carcinomas (Jeffers et al, 1997; Schmidt et al, 1997), did not show any nucleotide alterations in HTh 83 and KAT-4 cells (data not shown). To investigate the possibility of covalent dimerization of receptors due to aberrant di-sulphide bond formation between cysteine residues in Met, we performed Western blot analysis under non-reducing conditions of immunoprecipitated Met. Covalent dimerization would result in a Met species of approximately 380 kDa; however, this was not detected in HTh 83 or KAT-4 (data not shown).

© Cancer Research Campaign 1999 

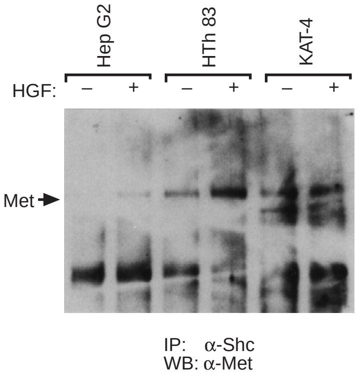

**Figure 8** Co-immunoprecipitation of Shc and Met in HTh 83 and KAT-4 cell lines. Total cell lysates were incubated with anti-Shc antibodies and GammaBind Plus Sepharose as described in Materials and Methods. After the immunoprecipitation and repeated washes the samples were boiled and electrophoresed under reducing conditions, followed by an immunoblot with the anti-Met antibody

### Co-immunoprecipitation of Met and Shc proteins

To demonstrate a functional coupling to intracellular signalling proteins of the constitutively phosphorylated receptors, we performed co-immunoprecipitation experiments with anti-Shc antibodies. As seen in Figure 8, binding of Met to Shc was detected in lysates from both unstimulated and HGF stimulated cells in the HTh 83 and KAT-4 cells. However, in the control cells Hep G2, co-precipitated Met was only seen after HGF stimulation (Figure 8). Furthermore, we could also precipitate Met in unstimulated KAT-4 cells using GST-fusion proteins with SH2-domains from PLC-γ and Grb2 (data not shown). Thus, it seems as if the constitutively phosphorylated receptors in the carcinoma cells are functionally active in signal transduction.

### DISCUSSION

Overexpression of Met has been observed in many different kinds of tumours, and there appears to be a link between Met activation, neoplastic transformation and malignant behaviour of tumour cells (Rong et al, 1992, 1994; Scotlandi et al, 1996). Anaplastic (undifferentiated) thyroid carcinoma of the giant cell type is one of the most aggressive and malignant tumours found in humans. The tumour usually kills the patient within a year from diagnosis (Carcangiu et al, 1985). In vivo, normal thyroid tissue barely expresses Met protein but many thyroid carcinomas have a 100-fold increase in Met expression (Di Renzo et al, 1991, 1992, 1995*b*). Interestingly, none of the anaplastic thyroid carcinomas studied ($n$ = 5) by Di Renzo et al (1995*b*) had an elevated Met expression, although, in the same study three out of four poorly differentiated thyroid carcinomas overexpressed Met. However, in the present study we found Met expression in all human anaplastic thyroid carcinoma cell lines studied. Interestingly, in four out of six cell lines the receptors were found to be tyrosine phosphorylated even in unstimulated cells, suggesting a constitutive activation of the receptors. This notion was strengthened by the finding of an association of Met to the adaptor protein Shc in unstimulated cells, indicating a functionally active receptor. Thus, our current data clearly show that some anaplastic thyroid carcinomas have the capacity to overexpress a constitutively activated Met protein.

Since it is known from other cell types that growth in vitro up-regulates Met expression (de Juan et al, 1994), we compared the expression level in the anaplastic thyroid carcinoma cells to the level found in primary cultures of normal human thyroid follicle cells. The expression of Met was severalfold higher in some of the thyroid carcinoma cell lines, compared to control cultures of normal thyrocytes, clearly showing an overexpression of Met.

Constitutive activation of Met, as observed in the present study, has previously been reported from a gastric carcinoma cell line (GTL-16) (Ponzetto et al, 1991), and also in murine melanoma cells (B16-LS9) (Rusciano et al, 1996). GTL-16 cells overexpress Met as a result of *MET* gene amplification (Ponzetto et al, 1991). So far, the exact mechanism behind the constitutive activation of Met in the GTL-16 cells has not been revealed; however, it is neither the result of rearrangements nor mutations of the *MET* gene (Ponzetto et al, 1991). A consistent finding in the present and previous studies is that a ligand-independent activation of Met is only observed in cell lines where Met is overexpressed (Ponzetto et al, 1991). Thus, it is possible that an overexpression leads to constitutive activation of the receptor proteins. Rusciano et al (1996) suggested that the ligand-independent activation observed in the B16-LS9 cells may be caused by the high number of receptors present on the cell surface, leading to spontaneous dimerization and phosphorylation. However, Ferracini et al (1995) reported very high expression of Met in the osteosarcoma cell line, U-2 OS, without any constitutive phosphorylation of the receptor.

Activation of the proto-oncogenes *ras* and *ret* is a common finding in thyroid carcinomas. Recently, Ivan et al (1997) demonstrated that infection of human thyrocytes with retroviruses containing activated *ras* or *ret* genes led to an up-regulation of the Met protein level. However, only one of the carcinoma cell lines in the present study, C 643, had activating *ras* mutations (Gly13Arg, in Ha-*ras* and Ala59Thr in Ki-*ras*; Ekwall et al, unpublished results) and none expressed activated *ret* (Alemi et al, unpublished results), suggesting that activation of *ras* or *ret* is not involved in the constitutive activation of Met.

Upon nucleotide sequencing of the Met kinase domain we were unable to detect any mutations in KAT-4 and HTh 83, showing that mutational activation of Met seems not to be present in our cell lines. Mutations in the kinase domain of Met in hereditary and sporadic papillary renal carcinomas were recently reported by Schmidt et al (1997). The mutations were located in the same region as activating mutations found in the Ret and Kit tyrosine kinase receptors, and were suggested to activate Met. The activating effect of the mutations was later demonstrated by Jeffers et al (1997). Neither did the HTh 83 or KAT-4 cell lines express a covalently dimerized receptor species, as judged by Western blot analysis under non-reducing conditions. This is the case in some of the mutated forms of Ret, where mutations in the cysteine-rich part of the extracellular domain yields a covalent association of receptors due to formation of di-sulphide bonds (Santoro et al, 1995).

Receptor phosphorylation and activation could be the result of an autocrine stimulation by HGF. Simultaneous expression of HGF and Met has been found in lung carcinoma cells (Tsao et al, 1993), in osteosarcoma cell lines (Ferracini et al, 1995), myeloma cell lines (Börset et al, 1996) and human breast carcinomas (Tuck et al, 1996). In the thyroid carcinoma cell lines in the present study we also found HGF mRNA expression in one of the cell lines (HTh 74). However, in this cell line we have so far been unable to demonstrate the existence of an autocrine activation of Met. The

 © Cancer Research Campaign 1999

cell lines showing constitutive activation of Met did not express HGF mRNA. Moreover, autocrine activation of Met in the thyroid carcinoma cells does not seem likely since preincubation with suramin, which blocks the interaction between HGF and Met (Adams et al, 1991; Figure 6), had no effect on the phosphorylation pattern of the receptors, thus further strengthening the conclusion that ligand independent activation of the receptor occurred.

Differences in phosphatase activity, maybe of the protein phosphatase functionally coupled to Met (Villa-Moruzzi et al, 1993; Fixman et al, 1996), could result in an increased fraction of phosphorylated Met receptors. Increased phosphatase activity upon detachment of cells from the substratum leading to a decreased Met phosphorylation has been observed in murine melanoma cells (Rusciano et al, 1996). The cell lines in our study express equal amounts of the phosphatase Syp 2, and so far we have not been able to detect any differences in the activity of the cellular phosphatases. Thus, the constitutive activity of Met is not likely to be the result of impaired activity of the cellular phosphatases.

The role of the constitutive activation and overexpression of Met observed in the anaplastic thyroid carcinoma cells is not known. So far we have been unable to detect any differences between cells with and without constitutive Met activation with respect to growth characteristics or migration (data not shown). However, we speculate that constitutive activation of Met might have been of importance in early steps of malignant transformation, but at present stage, the contribution to the malignant phenotype is limited due to additional transformational events that these highly malignant tumour cells have undergone.

In summary, Met is expressed in all human anaplastic thyroid carcinoma cell lines studied. In four carcinomas with high expression levels of Met there was a ligand-independent activation of the receptors. The mechanism(s) behind the constitutive receptor phosphorylation and activation is not clear; however, receptor number seems to be an important factor. The importance of the overexpression and constitutive activation of Met for the malignant phenotype of the anaplastic thyroid carcinoma cells remains to be elucidated.

### ACKNOWLEDGEMENTS

This work was supported by the Swedish Medical Research Council (Project no 11207). The authors wish to thank Dr Kenneth B Ain for providing the KAT-4 cell line.

### REFERENCES

Adams JC, Furlong RA and Watt FM (1991) Production of scatter factor by ndk, a strain of epithelial cells, and inhibition of scatter factor activity by suramin. *J Cell Sci* **98**: 385–394

Ain KB and Taylor KD (1994) Somatostatin analogs affects proliferation of human thyroid carcinoma cell lines in vitro. *J Clin Endocrinol Metab* **78**: 1097–1102

Auffray C and Rougeon F (1980) Purification of mouse immunoglobulin heavy-chain messenger RNAs from total myeloma tumor RNA. *Eur J Biochem* **107**: 303–314

Bardelli A, Maina F, Gout I, Fry MJ, Waterfield MD, Comoglio PM and Ponzetto C (1992) Autophosphorylation promotes complex formation of recombinant hepatocyte growth factor receptor with cytoplasmic effectors containing SH2 domains. *Oncogene* **7**: 1973–1978

Bardelli A, Ponzetto C and Comoglio PM (1994) Identification of functional domains in the hepatocyte growth factor and its receptor by molecular engineering. *J Biotechnol* **37**: 109–122

Bottaro DP, Rubin JS, Faletto DL, Chan AM-L, Kmiecik TE, Vande Woude GF and Aaronson SA (1991) Identification of the hepatocyte growth factor receptor as the *c-met* proto-oncogene product. *Science* **251**: 802–804

Börset M, Lien E, Espevik T, Helseth E, Waage A and Sundan A (1996) Concomitant expression of hepatocyte growth factor/scatter factor and its receptor in human myeloma cell lines. *J Biol Chem* **271**: 24655–24661

Carlsson J, Nilsson K, Westermark B, Pontén J, Sundström C, Larsson E, Bergh J, Påhlman S, Busch C and Collins VP (1983) Formation and growth of multicellular spheroids of human origin. *Int J Cancer* **31**: 523–533

Carcangiu ML, Steeper T, Zampi G and Rosai J (1985) Anaplastic thyroid carcinoma. A study of 70 cases. *Am J Clin Pathol* **83**: 135–158

de Juan C, Sánchez A, Nakamura T, Fabregat I and Benito M (1994) Hepatocyte growth factor up-regulates met expression in rat fetal hepatocytes in primary culture. *Biochem Biophys Res Commun* **204**: 1364–1370

Di Renzo MF, Narsimhan RP, Olivero M, Bretti S, Giordano S, Medico E, Gaglio P, Zara P and Comoglio PM (1991) Expression of the Met/HGF receptor in normal and neoplastic human tissues. *Oncogene* **6**: 1997–2003

Di Renzo MF, Olivero M, Ferro S, Prat M, Bongarzone I, Pilotti S, Belfiore A, Costantino A, Vigneri R, Pierotti MA and Comoglio PM (1992) Overexpression of the c-MET/HGF receptor gene in human thyroid carcinomas. *Oncogene* **7**: 2549–2553

Di Renzo MF, Olivero M, Katsaros D, Crepaldi T, Gaglia P, Zola P, Sismondi P and Comoglio PM (1994) Overexpression of the Met/HGF receptor in ovarian cancer. *Int J Cancer* **58**: 658–662

Di Renzo MF, Poulsom R, Olivero M, Comoglio PM and Lemoine NR (1995*a*) Expression of the Met/hepatocyte growth factor receptor in human pancreatic cancer. *Cancer Res* **55**: 1129–1138

Di Renzo MF, Olivero M, Serini G, Orlandi F, Pilotti S, Belfiori A, Costantino A, Vigneri R, Angeli A, Pierotti MA and Comoglio PM (1995*b*) Overexpression of the c-MET/HGF receptor in human thyroid carcinomas derived from the follicular epithelium. *J Endocrinol Invest* **18**: 134–139

Dremier S, Taton M, Coulonval K, Nakamura T, Matsumoto K and Dumont JE (1994) Mitogenic, de-differentiating, and scattering effects of hepatocyte growth factor on dog thyroid cells. *Endocrinology* **135**: 135–140

Eccles N, Ivan M and Wynford-Thomas D (1996) Mitogenic stimulation of normal and oncogene-transformed human thyroid epithelial cells by hepatocyte growth factor. *Mol Cell Endocrinol* **117**: 247–251

Ferracini R, Di Renzo MF, Scotlandi K, Baldini N, Olivero M, Lollini PL, Cremona O, Campanacci M and Comoglio PM (1995) The Met/HGF paracrine or an autocrine circuit. *Oncogene* **10**: 739–749

Fixman ED, Fournier TM, Kamikura DM, Naujokas MA and Park M (1996) Pathways downstream of Shc and Grb2 are required for cell transformation by the Tpr–Met oncoprotein. *J Biol Chem* **271**: 13116–13122

Gambarotta G, Boccacio C, Giordano S, Ando M, Stella MC and Comoglio PM (1996) *Ets* up-regulates *MET* transcription. *Oncogene* **13**: 1911–1917

Giordano S, Di Renzo MF, Narsimhan RP, Cooper CS, Rosa C and Comoglio PM (1989) Biosynthesis of the protein encoded by the *c-met* proto-oncogene. *Oncogene* **4**: 1383–1388

Heldin N-E and Westermark B (1988) Epidermal growth factor, but not thyrotropin, stimulates the expression of c-*fos* and c-*myc* messenger ribonucleic acid in porcine thyroid follicle cells in primary culture. *Endocrinology* **122**: 1042–1046

Heldin N-E, Cvejic D, Smeds S and Westermark B (1991) Coexpression of functionally active receptors for thyrotopin and platelet derived growth factor in human thyroid carcinoma cells. *Endocrinology* **129**: 2187–2193

Heldin N-E, Gustavsson B, Claesson-Welsh L, Hammacher A, Mark J, Heldin C-H and Westermark B (1988) Aberrant expression of receptors for platelet derived growth factor in an anaplastic thyroid carcinoma cell line. *Proc Natl Acad Sci USA* **85**: 9302–9306

Higashio K, Shima N, Goto M, Itagaki Y, Nagao M, Yasuda H and Morinaga T (1990) Identity of a tumor cytotoxic factor from human fibroblasts and hepatocyte growth factor. *Bichem Biophys Res Commun* **170**: 397–404

Ivan M, Bond JA, Prat M, Comoglio PM and Wynford-Thomas D (1997) Activated ras and ret oncogenes induce overexpression of c-met (hepatocyte growth factor receptor) in human thyroid epithelial cells. *Oncogene* **14**: 2417–2423

Jeffers M, Schmidt L, Nakaigawa N, Webb CP, Weirich G, Kishida T, Zbar B and Vande Woude GF (1997) Activating mutations for the Met tyrosine kinase receptor in human cancer. *Proc Natl Acad Sci USA* **94**: 11445–11450

Longati P, Bardelli A, Ponzetto C, Naldini L and Comoglio PM (1994) Tyrosines$^{1234–1235}$ are critical for the activation of the tyrosine kinase encoded by the MET proto-oncogene (HGF receptor). *Oncogene* **9**: 49–57

Naldini L, Weidner KM, Vigna E, Guadino G, Bardelli A, Ponzetto C, Narsimham RP, Hartmann G, Zarnegar R, Michalopoulos GK, Birchmeier W and Comoglio

© Cancer Research Campaign 1999

PM (1991) Scatter factor and hepatocyte growth factor are indistinguishable ligands for the MET receptor. *EMBO J* **10**: 2867–2878

Nakamura T, Nishizawa T, Hagiya M, Seki T, Shimonishi M, Sugimura A, Tashiro K and Shimizu S (1989) Molecular cloning and expression of human hepatocyte growth factor. *Nature* **342**: 440–443

Ponzetto C, Bardelli A, Zhen Z, Maina F, dalla Zonca P, Giordano S, Graziani A, Panayotou G and Comoglio PM (1994) A multifunctional docking site mediates signaling and transformation by the hepatocyte growth factor/scatter factor receptor family. *Cell* **77**: 261–271

Ponzetto C, Giordano S, Peverali F, Della Valle G, Abate M, Vaula G and Comoglio PM (1991) c-*met* is amplified but not mutated in a cell line with an activated met tyrosine kinase. *Oncogene* **6**: 553–559

Prat M, Narsimhan R, Crepaldi T, Nicotra R, Natali PG and Comoglio PM (1991) The receptor encoded by the human c-met oncogene is expressed in hepatocytes, epithelial cells and solid tumors. *Int J Cancer* **49**: 323–328

Rong S, Bodescot M, Blair D, Dunn J, Nakamura T, Mizuno K, Park M, Chan A, Aaronson S and Vande Woude GF (1992) Tumorigenicity of the *met* proto-oncogene and the gene for hepatocyte growth factor. *Mol Cell Biol* **12**: 5152–5158

Rong S, Segal S, Anver M, Resau JH and Vande Woude GF (1994) Invasiveness and metastasis of NIH 3T3 cells induced by Met-hepatocyte growth factor/scatter factor autocrine stimulation. *Proc Natl Acad Sci USA* **91**: 4731–4735

Rubin JS, Chan AM, Bottaro DP, Burgess WH, Taylor WG, Cech AC, Hirschfield DW, Wong J, Miki T, Finch PW and Aaronsson SA (1991) A broad-spectrum human lung fibroblast-derived mitogen is a variant of hepatocyte growth factor. *Proc Natl Acad Sci USA* **88**: 415–419

Rusciano D, Lorenzoni P and Burger MM (1996) Constitutive activation of c-Met in liver metastatic B16 melanoma cells depends on both substrate adhesion and cell density and is regulated by a cytosolic tyrosine phosphatase activity. *J Biol Chem* **271**: 20763–20769

Santoro M, Carlomagno F, Romano A, Bottaro DP, Dathan NA, Grieco M, Fusco A, Vecchio G, Matoskova B, Kraus MH and Di Fiore PP (1995) Activation of Ret as a dominant transforming gene by germline mutations of MEN 2A and MEN 2B. *Science* **267**: 381–383

Schmidt L, Duh FM, Chen F, Kishida T, Glenn G, Choyke P, Scherer SW, Zhuang Z, Lubensky I, Dean M, Allikmets R, Chidambaram A, Bergerheim UR, Feltis JT, Casadevall C, Zamarron A, Bernues M, Richard S, Lips CJ, Walther MM, Tsui LC, Geil L, Orcutt ML, Stackhouse T, Lipan J, Slife L, Brauch H, Decker J, Niehans G, Hughson MD, Moch H, Storkel S, Lerman MI, Linehan WM and Zbar B (1997) Germline and somatic mutations in the tyrosine kinase domain of the MET proto-oncogene in papillary renal carcinomas. *Nat Genet* **16**: 68–73

Scotlandi K, Baldini N, Oliviero M, Di Renzo MF, Martana M, Manara MC, Comoglio PM and Ferracini R (1996) Expression of Met/hepatocyte growth factor receptor gene and malignant behavior of musculoskeletal tumors. *Am J Pathol* **149**: 1209–1219

Stoker M, Gherardi E and Gray J (1987) Scatter factor is a fibroblast-derived modulator of epithelial cell mobility. *Nature* **327**: 239–242

Tsao M-S, Zhu H, Giaid A, Viallet J, Nakamura T and Park M (1993) Hepatocyte growth factor/scatter factor is an autocrine factor for human normal bronchial epithelial and lung carcinoma cells. *Cell Growth & Diff* **4**: 571–579

Tso JY, Sun XH, Kao T, Reece KS and Wu R (1985) Isolation and characterization of rat and human glyceraldehyde-3-phosphate dehydrogenase cDNAs: genomic complexity and molecular evolution of the gene. *Nucleic Acids Res* **13**: 2485–2502

Tuck AB, Park M, Sterns EE, Boag A and Elliott BE (1996) Coexpression of hepatocyte growth factor and receptor (Met) in human breast carcinoma. *Am J Pathol* **148**: 225–232.

Villa-Moruzzi E, Lapi S, Prat M, Gaudino G and Comoglio PM (1993) A protein tyrosine phosphatase activity associated with the hepatocyte growth factor/scatter factor receptor. *J Biol Chem* **268**: 18176–18180

Weidner KM, Arakaki N, Hartmann G, Vandekerckhove J, Weingart S, Rieder H, Fonatsch C, Tsubouchi H, Hishida T, Daikuhara Y and Birchmeier W (1991) Evidence for the identity of human scatter factor and human hepatocyte growth factor. *Proc Natl Acad Sci USA* **88**: 7001–7005

Weidner KM, Di Cesare S, Sachs M, Brinkmann V, Behrens J and Birchmeier W (1996) Interaction between Gab1 and the c-Met receptor tyrosine kinase is responsible for epithelial morphogenesis. *Nature* **384**: 173–176

Westermark K, Karlsson FA and Westermark B (1983) Epidermal growth factor modulates thyroid growth and function in culture. *Endocrinology* **112**: 1680–1686

Zarnegar R and Michalopoulos GK (1995) The many faces of hepatocyte growth factor: from hepatopoiesis to hematopoiesis. *J Cell Biol* **129**: 1177–1180

Zarnegar R, Muga S, Rahija R and Michalopoulos G (1990) Tissue distribution of hepatopoietin-A: a heparin-binding polypeptide growth factor for hepatocytes. *Proc Natl Acad Sci USA* **87**: 1252–1256

Zhen Z, Giordano S, Longati P, Medico E, Campiglio M and Comoglio PM (1994) Structural and functional domains critical for constitutive activation of the HGF-receptor. *Oncogene* **9**: 1691–1697

*© Cancer Research Campaign 1999*
